# The impact of the COVID‐19 pandemic on perioperative chemotherapy for breast cancer

**DOI:** 10.1002/cam4.5898

**Published:** 2023-04-03

**Authors:** Kaede Baba, Megumi Kawamoto, Kanako Mamishin, Mao Uematsu, Hikari Kiyohara, Akira Hirota, Nobuyuki Takahashi, Misao Fukuda, Shota Kusuhara, Hiromichi Nakajima, Chikako Funasaka, Takehiro Nakao, Chihiro Kondoh, Kenichi Harano, Nobuaki Matsubara, Yoichi Naito, Ako Hosono, Toshikatsu Kawasaki, Toru Mukohara

**Affiliations:** ^1^ Department of Pharmacy National Cancer Center Hospital East 6‐5‐1 Kashiwanoha Kashiwa 277‐8577 Japan; ^2^ Department of Medical Oncology National Cancer Center Hospital East 6‐5‐1 Kashiwanoha Kashiwa 277‐8577 Japan; ^3^ Department of Experimental Therapeutics National Cancer Center Hospital East 6‐5‐1 Kashiwanoha Kashiwa 277‐8577 Japan; ^4^ Department of General Internal Medicine National Cancer Center Hospital East 6‐5‐1 Kashiwanoha Kashiwa 277‐8577 Japan; ^5^ Department of Pediatric Oncology National Cancer Center Hospital East 6‐5‐1 Kashiwanoha Kashiwa 277‐8577 Japan

**Keywords:** adjuvant chemotherapy, breast cancer, COVID‐19, neoadjuvant chemotherapy, SARS‐CoV‐2

## Abstract

**Background:**

Since it was first reported in December 2019, coronavirus disease 2019 (COVID‐19) spread rapidly across the globe resulting in a pandemic. As of August 2022, seven outbreak peaks have been confirmed in Tokyo, and the numbers of new cases in the fifth and later outbreak periods have been far greater than in the preceding periods. This retrospective study examined the impact of the COVID‐19 pandemic on perioperative chemotherapy for breast cancer.

**Methods:**

Patients with breast cancer who received perioperative chemotherapy at the National Cancer Center Hospital East were divided into 2 groups: 120 and 384 patients who started chemotherapy before and during the pandemic, respectively. The incidence of critical events that had potential detrimental effects on the prognosis, such as start of adjuvant chemotherapy ≥91 days after surgery and relative dose intensity of chemotherapy <85% were compared between groups.

**Results:**

No significant difference in the incidence of critical events was found. When stratified by outbreak period, the incidence of critical events was positively correlated with the increasing number of new cases of COVID‐19 (*r* = 0.83, *p* = 0.04). Moreover, 25/173 patients (14%) who started perioperative chemotherapy during the fifth and sixth outbreak periods developed COVID‐19 infection, 80% of whom (20/25) had a delay or interruption to their surgery or other perioperative treatments.

**Conclusions:**

Although the impact of the COVID‐19 pandemic on perioperative chemotherapy on whole groups of patients was not evident when comparing periods before and after the pandemic, the impact is becoming prominent in parallel with increasing numbers of new COVID‐19 cases.

## Graphical abstract

This retrospective study examined the impact of the COVID‐19 pandemic on perioperative chemotherapy for breast cancer. Patients with breast cancer who received perioperative chemotherapy were divided into 2 groups: 120 and 384 patients who started chemotherapy before and during the pandemic, respectively. Although no significant difference in the incidence of critical events that had potential detrimental effects on the prognosis, such as start of adjuvant chemotherapy ≥91 days after surgery and relative dose intensity of chemotherapy <85% was found, the incidence tended to increase through the outbreak periods, particularly the fifth and sixth periods.

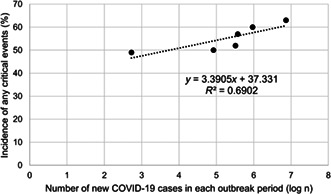

## 1 INTRODUCTION

Coronavirus disease 2019 (COVID‐19) is a respiratory tract infection caused by severe acute respiratory syndrome coronavirus 2 (SARS‐CoV‐2) infection, which was first reported in China in December 2019. By June 2022, more than 541 million people worldwide had been infected and more than 6.3 million had died, while in Japan, more than 9 million people had been infected and more than 30,000 had died.^1^ As of August 2022, seven outbreak peaks have been confirmed in the Tokyo metropolitan area, and the number of new cases in the fifth and later outbreak periods is more than three times that of the preceding periods (Figure S1).^1^ COVID‐19 infection presents with nonspecific symptoms such as fever, respiratory symptoms, fatigue, and headache. Most patients have mild disease, but approximately 5% require ventilator therapy, and death can occur in severe cases.^1^

Recently, it has become clear that the COVID‐19 pandemic has affected cancer patients in a variety of ways. As a direct effect, cancer patients appear to be more susceptible to COVID‐19, and COVID‐19 infection in cancer patients is a high risk for serious outcomes.^2^, ^3^ The COVID‐19 pandemic may have also affected cancer diagnosis and treatment, because medical facilities have to allocate more resources to care for COVID‐19 patients and cancer patients may avoid visiting medical facilities and receiving immune‐compromising treatment. Indeed, a report suggested a delay in diagnoses resulted in an increase in breast cancer tumor size and lymph node staging at initial presentation after the lockdown in Italy caused by the COVID‐19 pandemic.^4^ Another study reported that fewer patients were receiving neoadjuvant chemotherapy (NAC) than in the pre‐COVID‐19 era.^5^ In addition, 3.8% of all patients in a clinical trial of breast cancer discontinued NAC because of COVID‐19 infection.^6^ It has been estimated that a delay in diagnosis and change in treatment could increase the number of deaths up to 5 years after a diagnosis of breast cancer by 7.9%–9.6% compared with the pre‐COVID‐19 pandemic level.^7^

Because the maintenance of relative dose intensity (RDI) is important in perioperative chemotherapy for breast cancer to maximally reduce the risk of recurrence,^8^ there are concerns about the negative impact of the COVID‐19 pandemic on treatment planning. The Japan Society of Clinical Oncology (JSCO), Japanese Cancer Association (JCA), and Japan Society of Medical Oncology (JSMO) issued the statement^9^ that perioperative chemotherapy during the COVID‐19 pandemic should adhere to the dosing schedule whenever possible while taking the estimated benefit of the treatment into account. The JSCO/JCA/JSMO statement also recommended the consideration of delaying the start of chemotherapy and selecting an adjuvant chemotherapy (Adj) regimen with lower bone marrow suppression effects for patients who are not at high risk of relapse. However, it is not clear how these statements affect real‐world clinical practice.

In addition to the COVID‐19 infection itself, vaccination against COVID‐19 may have affected clinical practice for breast cancer. In Japan, two types of vaccines were preferentially administered to potentially immune‐compromised people including patients with cancer, elderly people, and healthcare workers. It was reported that the frequency of adverse reactions caused by COVID‐19 vaccination in cancer patients was in general comparable with that in healthy adults, and there should be no safety issues.^10^, ^11^ Conversely, symptoms such as fever and fatigue are similar to the side effects of chemotherapy. In particular, fever after COVID‐19 vaccinations during perioperative chemotherapy is difficult for patients as well as medical personnel to determine because it is similar to that caused by febrile neutropenia; therefore COVID‐19 vaccinations may have affected various clinical practices including changes in the dose and schedule of perioperative chemotherapy.

Although the COVID‐19 pandemic may have affected the implementation of perioperative therapy related to the infection itself or other related events such as vaccination and close contact, its impact on daily practice of chemotherapy is unknown. Therefore, we evaluated the impact of the COVID‐19 pandemic on perioperative chemotherapy for breast cancer in real‐world clinical practice in Japan.

## 2 MATERIALS AND METHODS

### 2.1 Study design and patients

**FIGURE 1:**
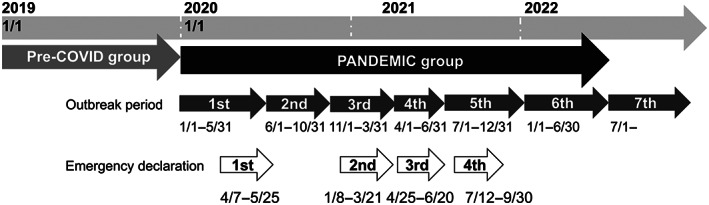
Data collection period. The periods covered by the pre‐COVID and PANDEMIC‐COVID groups are shown.

We retrospectively analyzed the medical records of breast cancer patients who started doxorubicin plus cyclophosphamide (AC) followed by a taxane (weekly paclitaxel [wPTX] or tri‐weekly docetaxel [DTX]), dose‐dense (dd)AC followed by a taxane (wPTX or DTX), or DTX plus cyclophosphamide (TC) as perioperative chemotherapy at National Cancer Center Hospital East in Chiba prefecture, Japan (Figure S2). The pre‐COVID‐19 group (PRE) consisted of patients who had started perioperative chemotherapy from January 1st, 2019 to December 31st, 2019, 4 months before the first emergency declaration for COVID‐19 was announced in Japan (Figure 1). The during‐COVID‐19 group (PANDEMIC) consisted of patients who started perioperative chemotherapy from January 1st, 2020 to June 30th, 2022 through six outbreak periods (Figures 1 and S1). The definition of each period was based on a document issued by an advisory board formed under the Ministry of Health, Labour and Welfare of Japan^12^: (i) January 2020 to May 2020; (ii) June 2020 to October 2020; (iii) November 2020 to March 2021; (iv) April 2021 to June 2021; (v) July 2021 to December 2021; and (vi) January 2022 to June 2022 (Figures 1 and S1). The period after the sixth outbreak period was tentatively defined as the seventh period in this study (Figures 1 and S1). Patients whose observation was terminated during the treatment and who started treatment for recurrent diseases were excluded. The date of data cutoff was November 30th, 2022.

### 2.2 Data collection and definition of detrimental events

Patient background data including age, sex, height, weight, Eastern Cooperative Oncology Group performance status (PS), hormone receptor status, human epidermal growth factor receptor‐2 (HER2) status, tumor size, and lymph node metastases were collected at the time when the first dose of perioperative chemotherapy was administered. For chemotherapy, information on the planned regimen, dose reduction, and withdrawal were collected until the end of perioperative chemotherapy. Information on pathological response (pathological complete response [pCR] or not) was also collected. Data detailing infection, close contact with COVID‐19 patients, and other narrative events related to the COVID‐19 pandemic, and their influence on surgery, perioperative chemotherapy, radiotherapy, or additional adjuvant therapies other than endocrine therapy, were also recorded.

The incidence of events that had a potentially detrimental effect on breast cancer prognosis was compared between the PRE and PANDEMIC groups. The critical events were defined as follows: delayed first visit to medical facilities for more than 3 months since recognizing the presence of a breast lump,^13^ RDI <85% in (dd)AC, a taxane, or TC,^14^ incompletion of all planned cycles of chemotherapy, postponement of two or more chemotherapy cycles,^14^ postponement of chemotherapy for 15 days or more,^14^ and 91 days or more elapsed between surgery and the start of Adj chemotherapy.^15^ These data were collected from the medical charts recorded from the date of the first visit to our hospital through to the date of the last administration of perioperative chemotherapy, which was defined as the primary observational period. Notable events were defined as follows: all death, and COVID‐19 infection during the entire observational period, which was defined as from the date of the first visit to our hospital to the data cutoff date.

### 2.3 Statistical analysis

Probability was calculated as a percentage, the *χ*2 test was used for the comparison of qualitative data, and the Mann–Whitney *U‐*test was used for the comparison of quantitative data. Pearson's correlation coefficient was used to test the relationship between continuous variables. *p* < 0.05 was considered statistically significant. Statistical data were analyzed using IBM SPSS Statistics 22.0.

### 2.4 Hospital location

The locations of the referral hospitals were plotted on a map of Japan. The map was created by processing the “Location Reference Information Download Service”^16^ (Ministry of Land, Infrastructure, Transport, and Tourism). The “CSV Address Matching Service”^17^ was used to plot the referral hospitals. These were created using Free and Open Source QGISv3.22.9 LTR.

### 2.5 Ethics

This study was approved by the Ethics Review Board of the National Cancer Center following the “Ethical Guidelines for Medical and Health Research Involving Human Subjects” (approval number: 2021–381). The data were anonymized and personal information was kept confidential.

## 3 RESULTS

### 3.1 Patient characteristics

Overall, 504 eligible patients, 120 in the PRE group and 384 in the PANDEMIC group, were identified. Patients' baseline characteristics are shown in Table 1. The median (range) of the primary observation period was 6 months (4–11 months) for the PRE group and 6 months (2–64 months) for the PANDEMIC group (Table 1). All patients were female, and the groups were balanced with regard to age, body surface area, PS, menstruation status, and subtypes. Clinical T stage or N stage at diagnosis was not significantly different between the PRE and PANDEMIC groups (Table 1).

**TABLE 1:** Patients' characteristics.

Baseline characteristic	PRE (*n* = 120)	PANDEMIC (*n* = 384)	*p‐*value
Entire observational period, months; median (range)	42 (7–49)	19 (4–67)	<0.001^a^
Primary observational period, months; median (range)	6 (4–11)	6 (2–64)	0.33^a^
Age, years, median (range)	53 (29–77)	54 (28–85)	0.80^a^
BMI, kg/m^2^; median (range)	23 (17–35)	23 (15–46)	0.17^a^
BSA, m^2^; median (range)	1.6 (1.2–2)	1.6 (1.2–2.2)	0.24^a^
Female, *n* (%)	120 (100)	384 (100)	‐
ECOG PS, *n* (%)			
0/1/unknown	110 (92)/3 (3)/7 (6)	335 (87)/28 (7)/21 (5)	0.06^b^
Presence of smoking history, *n* (%)	28 (23)	121 (31)	0.09^b^
Reason for consultation, *n* (%)			
Symptom/screening/others	79 (66)/28 (23)/13 (11)	275 (72)/81 (21)/28 (7)	0.47^b^
Menopause, *n* (%)			
Pre/post	54 (45)/65 (54)	168 (44)/211 (55)	0.77^b^
Subtype, *n* (%)			
HR (+)	66 (55)	245 (64)	0.08^b^
HER2 (+)	29 (24)	80 (21)	0.44^b^
TN	43 (36)	104 (27)	0.07^b^
Clinical T stage, *n* (%)			
T0/Tis/T1/T2/T3/T4/unknown	0 (0)/1 (1)/20 (17)/70 (58)/21 (18)/7 (6)/1 (1)	1 (0.3)/3 (0.8)/97 (25)/220 (57)/38 (10)/23 (6)/2 (0.5)	0.17^b^
Clinical N stage, *n* (%)			
N0/N1/N2/N3/unknown	55 (46)/46 (38)/3 (3)/14 (12)/2 (2)	218 (57)/121 (32)/6 (2)/35 (9)/4 (1)	0.22^b^

Abbreviations: BMI, body mass index; BSA, body surface area; ECOG PS, Eastern Cooperative Oncology Group Performance State; HR, hormone receptor; HER2, human epidermal growth factor receptor2; TN, triple negative.

^a^ Mann‐Whitney *U‐*test.

^b^ Chi‐square test.

The details of surgery and perioperative treatment are shown in Table 2. There was no significant difference in surgical procedures, with 84/119 (71%) patients in the PRE group and 232/359 (65%) patients in the PANDEMIC group undergoing mastectomy. Axillary lymph node resection tended to be more common in the PRE group, but this did not reach statistical significance (61% vs. 52%, *p* = 0.07). The proportion of Adj/NAC was generally 50% each in both groups. Even though an increase in ddAC in the PANDEMIC group was observed (PRE vs. PANDEMIC, 0 patients [0%] vs. 54 patients [14%]), this occurred because we resumed using ddAC in 2021 following an interruption since January 2018 because of concerns regarding its toxicity. This resumption was based on reports that ddAC is safe and well tolerated compared with AC.^18^ There was no difference in the selection of taxane, wPTX, or DTX, between the groups.

**TABLE 2:** Overview of surgery and perioperative therapy.

Perioperative therapy	PRE (*n* = 120)	PANDEMIC (*n* = 384)	*p‐*value^c^
Surgery, *n* (%)^a^			
Bp/Bt	35 (29)/84 (71)	127 (35)/232 (65)	0.23
Ax/no Ax	73 (61)/46 (39)	186 (52)/174 (48)	0.07
Radiotherapy, *n* (%)			0.24
Yes/No	94 (79)/25 (21)	262(75)/89 (25)	
Chemotherapy, *n* (%)^b^			
NAC/Adj	65 (54)/55 (46)	202 (53)/182 (47)	0.77
AC/ddAC/TC	101 (84)/0 (0)/19 (16)	246 (64)/54 (14)/84 (22)	<0.001
wPTX/DTX	31 (26)/69 (58)	111 (29)/181 (47)	0.21

Abbreviations: AC, doxorubicin + cyclophosphamide; Adj, adjuvant chemotherapy; Ax, axillary lymph node dissection; Bp, breast partial resection; Bt, breast total resection; DTX, tri‐weekly docetaxel; NAC, neoadjuvant chemotherapy; TC, docetaxel + cyclophosphamide; wPTX, weekly paclitaxel.

^a^ One patient in the PRE group died, and 24 patients of the PANDEMIC group did not undergo surgery for the following reasons: 11 patients were awaiting surgery at data cutoff, 8 patients participated in a clinical trial to omit surgery, 2 patients had distant metastases, and 1 each died, refused surgery, and canceled surgery because of another emerging cancer. One patient underwent axillary dissection only and no mastectomy.

^b^ Patients who changed to taxane during the course of the study were excluded.

^c^ Chi‐square test.

### 3.2 Critical events

Information on the critical events (see Data collection and definition of detrimental events) that might have compromised the efficacy of perioperative chemotherapy was collected from the date of the first visit to our hospital through to the date of the last administration of perioperative chemotherapy, which was defined as the primary observational period. Critical events occurred in a similar proportion of patients, 56% and 57% of the PRE and PANDEMIC groups, respectively (*p* = 0.86) (Table 3). When examining each event, a numerically higher proportion of patients required 91 or more days to start Adj in the PANDEMIC group but the difference did not reach statistical significance (PRE vs. PANDEMIC, 1/55 [2%] vs. 12/182 [7%], *p* = 0.17) (Table 3).

**TABLE 3:** Critical events.

Critical events	PRE (*n* = 120)		PANDEMIC (*n* = 384)		
	*n* (%)	Total, *n*	*n* (%)	Total, *n*	*p*‐value^a^
All critical events	67 (56)	120	218 (57)	384	0.86
RDI <85%	32 (27)	120	115 (30)	384	0.49
Time to the first visit ≥3 months^b^					
Symptom	27 (34)	79	109 (40)	275	0.37
Screening	3 (11)	28	3 (4)	81	0.16
Postponement ≥15 days	13 (11)	120	46 (12)	384	0.73
Postponement ≥2 cycles	33 (28)	120	78 (20)	384	0.10
Start of Adj ≥91 days after surgery	1 (2)	55	12 (7)	182	0.17

Abbreviations: Adj, adjuvant chemotherapy; RDI, relative dose intensity.

^a^ Chi‐square test.

^b^ Excluding patients who were diagnosed with breast cancer while under follow‐up for other cancers in our hospital.

Treatment incompletion occurred in 15/120 (13%) patients in the PRE group and 53/384 (12%) patients in the PANDEMIC group. These patients were excluded from the following analysis by dose intensity. The proportion of patients with an RDI <85% was not different between the groups (PRE vs. PANDEMIC, 27% vs. 30%, *p* = 0.49) (Table 3).

**FIGURE 2:**
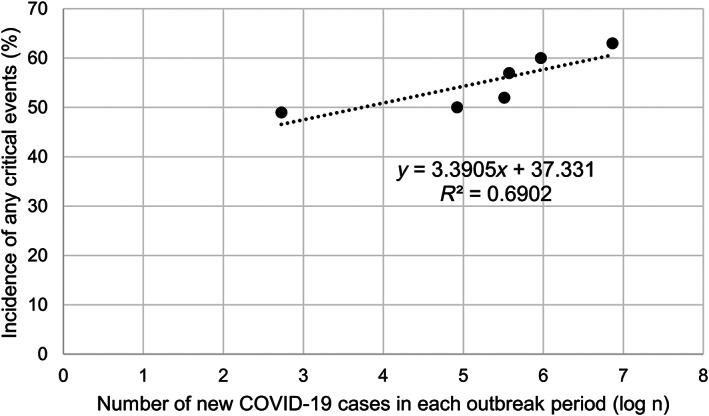
Correlation between the number of new cases of COVID‐19 and the incidence of critical events during each outbreak period. Pearson's correlation coefficient (*r*) was calculated with the logarithm of the number of new COVID‐19 cases in Japan and the incidence of any critical events, showing a positive correlation (*r* = 0.83, *p* = 0.04). The approximation formula and coefficient of determination (*R* ^2^) are shown in the figure.

When examining the incidence of these critical events by outbreak period, there was a tendency for an increase in the fifth and sixth periods (Table 4), when the number of new cases of COVID‐19 infection was more than three times those of the preceding periods (Figure S1). An exploratory analysis showed a positive correlation between the number of new cases of COVID‐19 in Japan and the incidence of any critical events in each outbreak (*r* = 0.83, *p* = 0.04) (Figure 2). Further stratified by each critical event, there appeared similar trends for chemotherapy postponement by ≥15 days, RDI <85%, and start of Adj ≥91 days after surgery (Figure S3). In particular, we noted that patients who started treatment after the fifth period (40%; 70/137) had an RDI <85% and 12% (10/83) required 91 or more days to start Adj (Table 4).

**TABLE 4:** Critical events in each period.

Critical events	1st	*n*	2nd	*n*	3rd	*n*	4th	*n*	5th	*n*	6th	*n*
All critical events	25 (49)	51	24 (50)	48	40 (57)	70	22 (52)	42	50 (60)	82	57 (63)	91
RDI < 85%, *n* (%)	10 (20)	51	6 (13)	48	19 (27)	70	10 (24)	42	28 (34)	82	42 (46)	91
Time to the first visit ≥3 months, *n* (%)^a^												
Symptom	12 (34)	35	19 (49)	39	21 (41)	51	15 (48)	31	19 (33)	58	23 (38)	61
Screening	1 (0.8)	13	0 (0)	7	1 (6)	17	0 (0)	9	0 (0)	15	1 (5)	20
Postponement ≥15 days, *n* (%)	8 (2)	51	4 (8)	48	6 (9)	70	2 (5)	42	12 (15)	82	14 (15)	91
Postponement ≥2 cycles, *n* (%)	9 (18)	51	9 (19)	48	19 (27)	70	3 (7)	42	22 (27)	82	16 (18)	91
Start of Adj ≥91 days after surgery	0 (0)	25	2 (4)	22	0 (0)	28	0 (0)	23	4 (10)	41	6 (14)	42

Abbreviations: Adj, adjuvant chemotherapy; RDI, relative dose intensity.

^a^ Excluding patients who were diagnosed with breast cancer while under follow‐up for other cancers in our hospital.

### 3.3 Notable events

**FIGURE 3:**
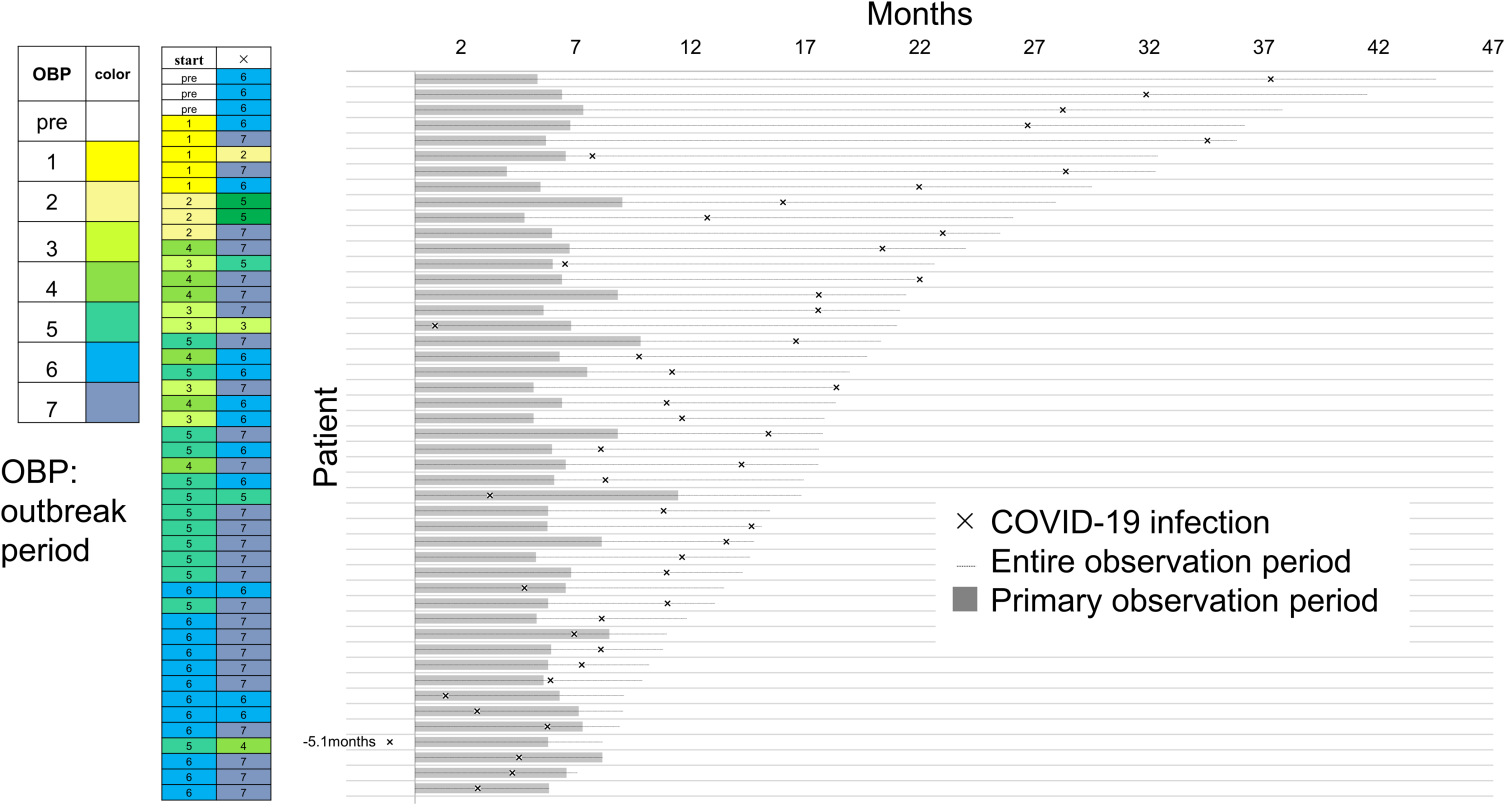
Details of the timing of COVID‐19 infection. Timing of COVID‐19 infection in each patient is shown in a swimmer plot. The left columns show outbreak periods, with “start” indicating the start of perioperative chemotherapy and “x” indicating the time of infection. See “Data collection and definition of detrimental events” for the definition of the observational period.

Information on notable events, that is, all death, and COVID‐19 infection, was collected during the entire observational period, which was defined as from the date of the first visit to our hospital to the data cutoff date (see Data collection and definition of detrimental events). There were two deaths in the PRE group and seven deaths in the PANDEMIC group. One death in the PRE group was caused by interstitial lung disease associated with chemotherapy and the other was caused by recurrent breast cancer. Two patients in the PANDEMIC group died from an unidentified cause and five died from recurrent breast cancer (Table 5). Over the entire observational period, COVID‐19 infection was observed in 3/120 (3%) patients in the PRE group and 44/384 (11%) patients in the PANDEMIC group (Table 5 and Figure 3). Notably, 26/47 cases occurred in the latest seventh outbreak period (Figure 3). No cases resulted in hospitalization or death related to COVID‐19 infection, but 27/384 (7%) patients in the PANDEMIC group required a postponement (delay or interruption) of surgery, perioperative chemotherapy, or additional postoperative therapy because of COVID‐19 infection (Table 6 upper row). Notably, 25/173 patients (14%) who started chemotherapy during the fifth or sixth outbreak period developed COVID‐19 infection, 80% of whom (20/25) had a delay or interruption of surgery, perioperative chemotherapy, or additional postoperative therapy related to COVID‐19 infection (Table 6 upper row). The impact of COVID‐19 related events other than infection, that is, close contact with an infected person, patient's avoidance of visiting hospital, and side effects of COVID‐19 vaccination, tended to increase the number of these events throughout the outbreak period (Table 6 lower row). Furthermore, 16/173 (9%) patients who started chemotherapy during the fifth or sixth outbreak period experienced COVID‐19 related events other than infection, 81% (13/16) of whom had a delay or interruption of surgery, perioperative chemotherapy, or additional postoperative therapy because of these event (Table 6).

**TABLE 5:** Notable events.

	PRE (*n* = 120)	PANDEMIC (*n* = 384)	*p‐*value^c^
	*n* (%)	*n* (%)	
All death	2 (2)^a^	7 (2)^a^	0.91
COVID‐19 infection	3 (3)	44 (11)	0.003

^a^ One patient in the PRE group died of interstitial lung disease associated with chemotherapy, and one of recurrent breast cancer. Two and five patients in the PANDEMIC group died from an unknown cause at home and of recurrent breast cancer, respectively.

**TABLE 6:** Impact of COVID‐19 pandemic in each period.

	1st (*n* = 51)	2nd (*n* = 48)	3rd (*n* = 70)	4th (*n* = 42)	5th (*n* = 82)	6th (*n* = 91)
COVID‐19 infection, *n* (%)						
Total	5 (10)	3 (6)	5 (7)	6 (14)	13 (16)	12 (13)
Postponement of Surgery	1 (2)	0 (0)	1 (1)	0 (0)	0 (0)	2 (2)
Postponement of perioperative chemotherapy^a^	0 (0)	0 (0)	0 (0)	0 (0)	1 (1)	8 (9)
Postponement of additional therapy^b^	0 (0)	1 (2)	2 (3)	3 (7)	8 (10)	1 (1)
No influence	4 (8)	2 (4)	2 (3)	3 (7)	4 (5)	1 (1)
Other COVID‐19 related events^c^, *n* (%)						
Total	2 (4)	2(4)	3 (4)	2 (5)	7 (9)	9 (10)
Postponement of Surgery	0 (0)	0 (0)	1 (1)	2 (5)	1 (1)	1 (1)
Postponement of perioperative chemotherapy^a^	0 (0)	2 (4)	0 (0)	0 (0)	4 (5)	5 (6)
Postponement of additional therapy^b^	0 (0)	0 (0)	2 (3)	0 (0)	2 (2)	0 (0)
No influence	2 (4)	0 (0)	0 (0)	0 (0)	0 (0)	3 (3)

^a^ Perioperative chemotherapy was defined as (dd)AC, TC, and taxane (wPTX and DTX).

^b^ Including radiotherapy and additional adjuvant chemotherapy (trastuzumab+pertuzumab, trastuzumab emtansine, abemaciclib, S‐1, and capecitabine).

^c^ Including close contact (12 patients), avoidance of visiting hospital (11 patients), and side effects to COVID‐19‐vaccinations (2 patients).

Despite this increasing trend throughout the outbreak periods, there was no difference in the pCR rate between the PRE and PANDEMIC groups (45% vs. 41%, *p* = 0.72) and no clear trend throughout the breakout periods (1st /2nd /3rd /4th /5th /6th; 42%, 35%, 46%, 44%, 41%, 37%).

### 3.4 Tendency of referral to hospital

To investigate the impact of COVID‐19 on regional medical resources, we examined information related to referral facilities. Details of the medical institutions from which the patients were referred are presented in Table S1. There was a 38% increase in referrals per year from clinics and a 21% increase from hospitals in the PANDEMIC group compared with the PRE group, although the ratio of clinics to hospitals before and after the COVID‐19 pandemic did not reach statistical significance (*p* = 0.53).

The median (range) distance from the referral facilities to our hospital was 7.8 km (1.1–432.4 km) for the PRE group and 8.0 km (1.1–1589 km) for the PANDEMIC group (Table S1). Although there was no significant difference in the distribution of prefectures where the referral hospitals were located between the PRE and PANDEMIC groups (*p* = 0.1) (Table S1), there was a numerical increase in the annual referral from facilities inside the same prefecture, that is, Chiba, in the PANDEMIC group (Table S1). An analysis of the distribution of the facilities on the map appeared to show an increase in referrals from areas close to Tokyo in the Saitama and Chiba prefectures (Figure S2).

## 4 DISCUSSION

In this study, we did not observe a significant difference in patient characteristics including tumor stage (Table 1) or treatment measures (Table 2) between the PRE and PANDEMIC groups. Also, factors potentially affecting the prognosis after perioperative chemotherapy for breast cancer, such as an RDI <85%, postponement of two or more treatment cycles, treatment postponement for 15 days or more, and 91 days or more elapsed between surgery and the start of chemotherapy did not differ significantly before or after the COVID‐19 pandemic (Table 3). However, patients who started perioperative chemotherapy during the fifth and sixth outbreak periods tended to be influenced by COVID‐19 infection and other related events more frequently than those who started treatment during preceding outbreak periods (Tables 4 and 6).

We initially expected that the screening rate might decline because of the COVID‐19 pandemic leading to an increase in the number of patients who were diagnosed with breast cancer with symptoms and at higher clinical stages, as reported in Italy.^4^ However, in our study, the proportion of patients diagnosed by screening and the tumor stage at diagnosis were similar between the PRE and PANDEMIC groups (Table 1). Three Japanese cancer screening facilities reported that the number of people screened decreased by 20%–80% from April to June 2020 compared with the same months in 2019, but recovered to the level of previous years from July 2020.^19^ Similarly, the Tokyo Metropolitan Bureau of Social Welfare and Public Health reported that breast cancer screening decreased by 36.7% in the first half of 2020 but increased to 102.4% in the second half.^20^ In addition, cancer screenings continued to be conducted under the second emergency declaration, activated on January 2021, with many municipalities extending the period available for the screening, which resulted in a year‐on‐year rate of 135.6% in March 2021. These reports indicate that although the first emergency declaration temporarily caused a significant drop in the screening rate, the number of people who received screening increased compensatively as the COVID‐19 pandemic became prolonged. For these reasons, the impact of the COVID‐19 pandemic on the time to initial consultation, disease stage at diagnosis, or invasiveness of surgical procedures may have been minor.

Similarly, our study showed no statistical difference between the PRE and PANDEMIC groups in critical events that might have detrimentally affected patients' prognoses. A breast cancer triage was developed by the Japanese Breast Cancer Society following the COVID‐19 pandemic in Japan. The triage followed the European Society for Medical Oncology guidelines that recommend giving high priority to the systemic treatment of early‐stage breast cancer in the following cases: chemotherapy for triple negative, HER2‐positive, high‐risk hormone receptor‐positive HER2‐negative breast cancers, and NAC that has already been started.^21^ Most of the patients in our study were categorized as high‐priority patients who required perioperative chemotherapy. In our hospital, strict measures to prevent the spread of COVID‐19 infection were actioned starting at the initial stage of the pandemic. These included body temperature screening for all who entered the hospital, and antigen or polymerase chain reaction testing for COVID‐19 on hospitalization. In addition, each prefecture established phase‐based anti‐COVID‐19 policies that set a number of reserved hospital beds according to each phase. Chiba prefecture activated four levels of the medical care provision system according to the phase: 1, 2A, 2B, and 3 (1, 2, 3, and 4 for the first few months of the pandemic), and our hospital provided a limited number of hospital beds to care for patients with COVID‐19 infection only when the phase reach 2B or higher. This environment likely minimized our contact with COVID‐19 patients and allowed us to concentrate on cancer care. Indeed, we did not experience a major COVID‐19 epidemic inside the hospital until the sixth outbreak period. Our current study indicates that we delivered appropriate chemotherapy to patients as planned during the COVID‐19 pandemic, which probably was a result of these efforts by our hospital and the regional medical facilities. Increase in the number of patient referrals during the COVID‐19 pandemic, particularly from areas close to Tokyo (Table S1 and Figure S2), may have resulted from our protected hospital environment while patients tended to avoid traveling to Tokyo, where COVID‐19 pandemic was remarkable.

The analysis of data by outbreak period suggests that the COVID‐19 pandemic affected our daily practice. After the fifth outbreak period, 14% of perioperative breast cancer patients were infected with COVID‐19, leading to treatment postponement in 80% of these patients (Table 6). Even more patients were affected when close contact and other COVID‐19 related events were included in the analysis (Table 6). The COVID‐19 pandemic expanded after the fifth outbreak period; in Chiba Prefecture, the number of infected people in the first four outbreak periods was in the 100 s per day, but in August 2021, the peak of the fifth outbreak period, 1000 to 2000 persons per day were reported, and in February 2022, the peak of the sixth wave, approximately 5000 persons per day were reported.^22^ Our current results indicate that the degree of impact on cancer treatment appears to parallel the degree of the spread of COVID‐19 infection. In addition, 11 cases of infection after the sixth outbreak period were observed in patients who started their perioperative chemotherapy during the fifth period. Of these, COVID‐19 infection in seven patients influenced their additional treatment following perioperative chemotherapy, such as delayed radiotherapy or adjuvant trastuzumab+pertuzumab therapy. Furthermore, one of the other four patients had a delay in the detection of de novo contralateral breast cancer because of the infection. These cases indicate that even if treatment was initiated at a time point after the peak of the outbreak, the repeat nature of the pandemic could still affect a patient's treatment. As such, the optimal time to start treatment and the sequence of treatments, e.g. NAC or Adj, are difficult to determine.

There were several limitations in this study. First, this was a retrospective study at a single institution. Our hospital is one of two national cancer‐specific hospitals, and our patients may have different backgrounds from those in other hospitals, limiting the generalizability of our results. Furthermore, it was difficult to determine whether patients in the PANDEMIC group were vaccinated, precluding the accurate judgment of how vaccination might affect treatment outcomes. Second, we resumed ddAC in 2021 after an interruption since pre‐pandemic 2018, which may have affected the RDI and other chemotherapy‐related data in a COVID‐19‐independent manner. However, the decision to resume ddAC was made based on our own data that the proportion of those who completed perioperative chemotherapy was equivalent between ddAC and conventional AC when both were followed by a taxane.^18^ Third, short follow‐up periods and the limited number of patients did not allow us to investigate the impact of the COVID‐19 pandemic on long‐term outcome measures such as disease‐free survival. Despite these limitations, we think that our results are important as a basis for considering breast cancer care under the COVID‐19 pandemic, which is expected to continue for some time.

In conclusion, our study showed that although the impact of the COVID‐19 pandemic on whole groups receiving perioperative chemotherapy for breast cancer was not evident when comparing periods before and after the pandemic, the impact is becoming more obvious in parallel with the increasing number of new cases of COVID‐19.

## AUTHOR CONTRIBUTIONS

**Kaede Baba:** Conceptualization (equal); data curation (lead); formal analysis (lead); investigation (lead); methodology (equal); validation (equal); visualization (lead); writing – original draft (equal); writing – review and editing (equal). **Megumi Kawamoto:** Conceptualization (equal); methodology (equal); writing – review and editing (equal). **Kanako Mamishin:** Conceptualization (equal); methodology (equal); writing – review and editing (equal). **Mao Uematsu:** Conceptualization (equal); methodology (equal); writing – review and editing (equal). **Hikari Kiyohara:** Conceptualization (equal); methodology (equal); writing – review and editing (equal). **Akira Hirota:** Conceptualization (equal); methodology (equal); writing – review and editing (equal). **Nobuyuki Takahashi:** Conceptualization (equal); methodology (equal); writing – review and editing (equal). **Misao Fukuda:** Conceptualization (equal); methodology (equal); writing – review and editing (equal). **Shota Kusuhara:** Conceptualization (equal); methodology (equal); writing – review and editing (equal). **Hiromichi Nakajima:** Conceptualization (equal); methodology (equal); writing – review and editing (equal). **Chikako Funasaka:** Conceptualization (equal); methodology (equal); writing – review and editing (equal). **Takehiro Nakao:** Conceptualization (equal); methodology (equal); writing – review and editing (equal). **Chihiro Kondoh:** Conceptualization (equal); methodology (equal); writing – review and editing (equal). **Kenichi Harano:** Conceptualization (equal); methodology (equal); writing – review and editing (equal). **Nobuaki Matsubara:** Conceptualization (equal); methodology (equal); writing – review and editing (equal). **Yoichi Naito:** Conceptualization (equal); methodology (equal); writing – review and editing (equal). **Ako Hosono:** Conceptualization (equal); methodology (equal); writing – review and editing (equal). **Toshikatsu Kawasaki:** Conceptualization (equal); methodology (equal); supervision (equal); writing – review and editing (equal). **Toru Mukohara:** Conceptualization (equal); investigation (equal); methodology (equal); supervision (equal); validation (equal); visualization (equal); writing – original draft (equal); writing – review and editing (equal).

## FUNDING INFORMATION

There is no funding involved.

## CONFLICT OF INTEREST STATEMENT

The authors have no conflict of interest.

## ETHICS STATEMENT

Approval of the research protocol by an Institutional Reviewer Board: This study was approved by the Ethics Review Board of the National Cancer Center following the “Ethical Guidelines for Medical and Health Research Involving Human Subjects” (approval number: 2021–381). Informed Consent: N/A. Registry and the Registration No. of the study/trial: N/A. Animal Studies: N/A.

## Supporting information


Table S1.

Figure S1.

Figure S2.

Figure S3.

## Data Availability

The data that support the findings of this study are available from the corresponding author upon reasonable request.
