# Supplementary material for: The impact of the COVID‐19 pandemic on perioperative chemotherapy for breast cancer
Source: Cancer Med. 2023 Apr 3;12(11):12095–105. doi: 10.1002/cam4.5898 (PMC10278517; doi:10.1002/cam4.5898)
Supplement: Supplementary file 1 — Table S1. [file CAM4-12-12095-s003.docx]

| Table S1. Details of previous medical institutions | | | | | |
| --- | --- | --- | --- | --- | --- |
| Details of previous medical institution | PRE (n=119) | | PANDEMIC (n=384) | | *P-*value |
| Clinics/hospitals, n (%) | 55 (46)/64 (54) | | 190 (49)/194 (51) | | 0.53^b^ |
| Distance to our hospital (km), median (range) | 7.8 (1.1-432.4) | | 8.0 (1.1-1589) | | 0.79^a^ |
| Number of referrals | Whole period | Per year | Whole period | Per year |  |
| Location, n (%)^c^  Within the same prefecture (Chiba)  Ibaraki  Saitama  Tokyo  Others | 78 (66)  16 (13)  12 (10)  12 (10)  1 (1) | 78  16  12  12  1 | 274 (71)  34 (9)  50 (13)  16 (4)  8 (2) | 110  14  20  6  3 | 0.10^a^ |
| ^a^Mann-Whitney *U-*test. ^b^Chi-square test. ^c^PRE indicates the number of referrals from each prefecture for the whole period (1 year) and PANDEMIC indicates the whole period (30 months) and for the calculated 1-year period. Cases of in-hospital consultations and consultations from abroad were excluded. | | | | | |
