# Supplementary figures and images for: The impact of the COVID‐19 pandemic on perioperative chemotherapy for breast cancer
Source: Cancer Med. 2023 Apr 3;12(11):12095–105. doi: 10.1002/cam4.5898 (PMC10278517; doi:10.1002/cam4.5898)

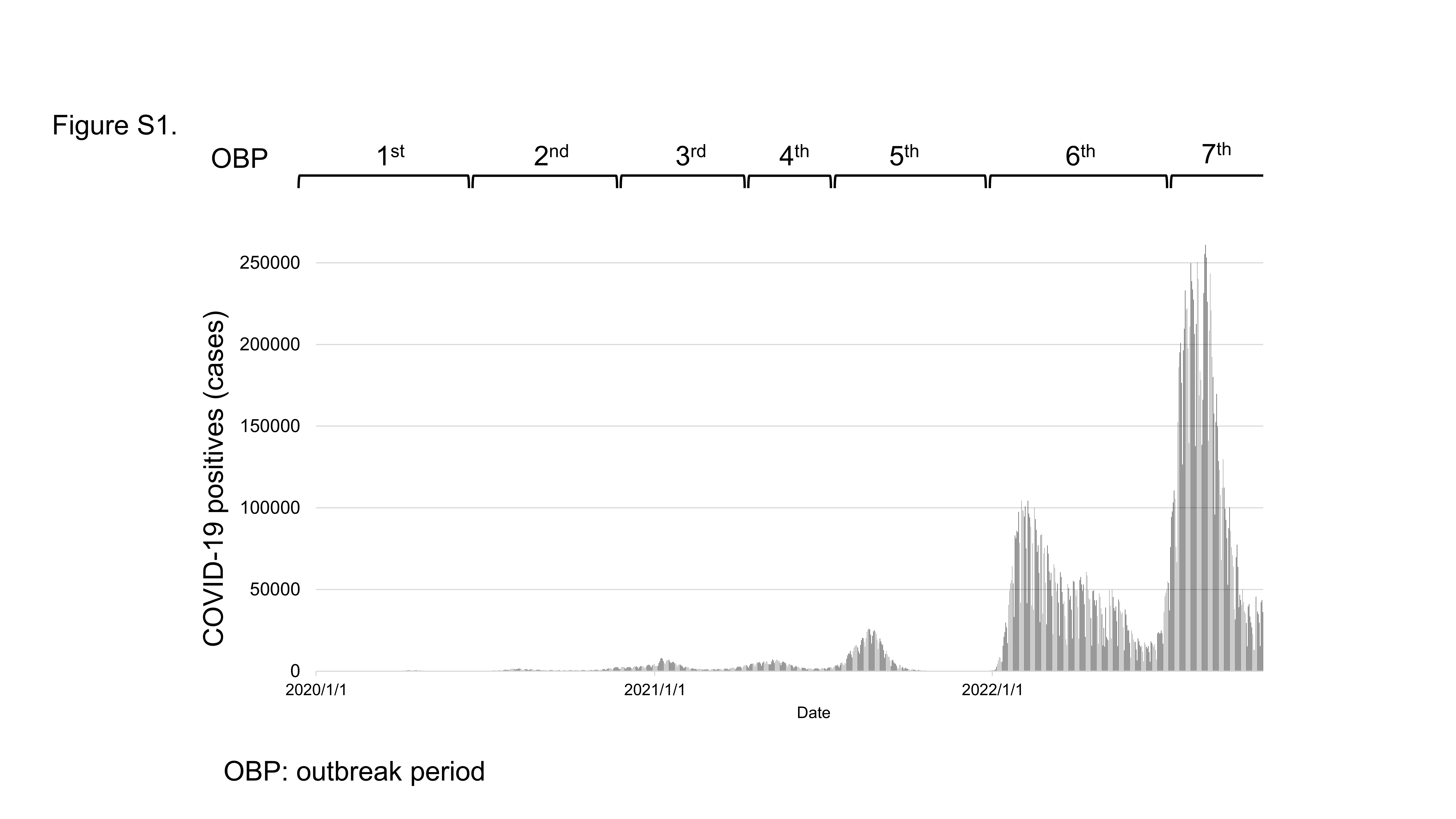

Supplement: Supplementary file 2 — Figure S1. [file CAM4-12-12095-s004.tif]

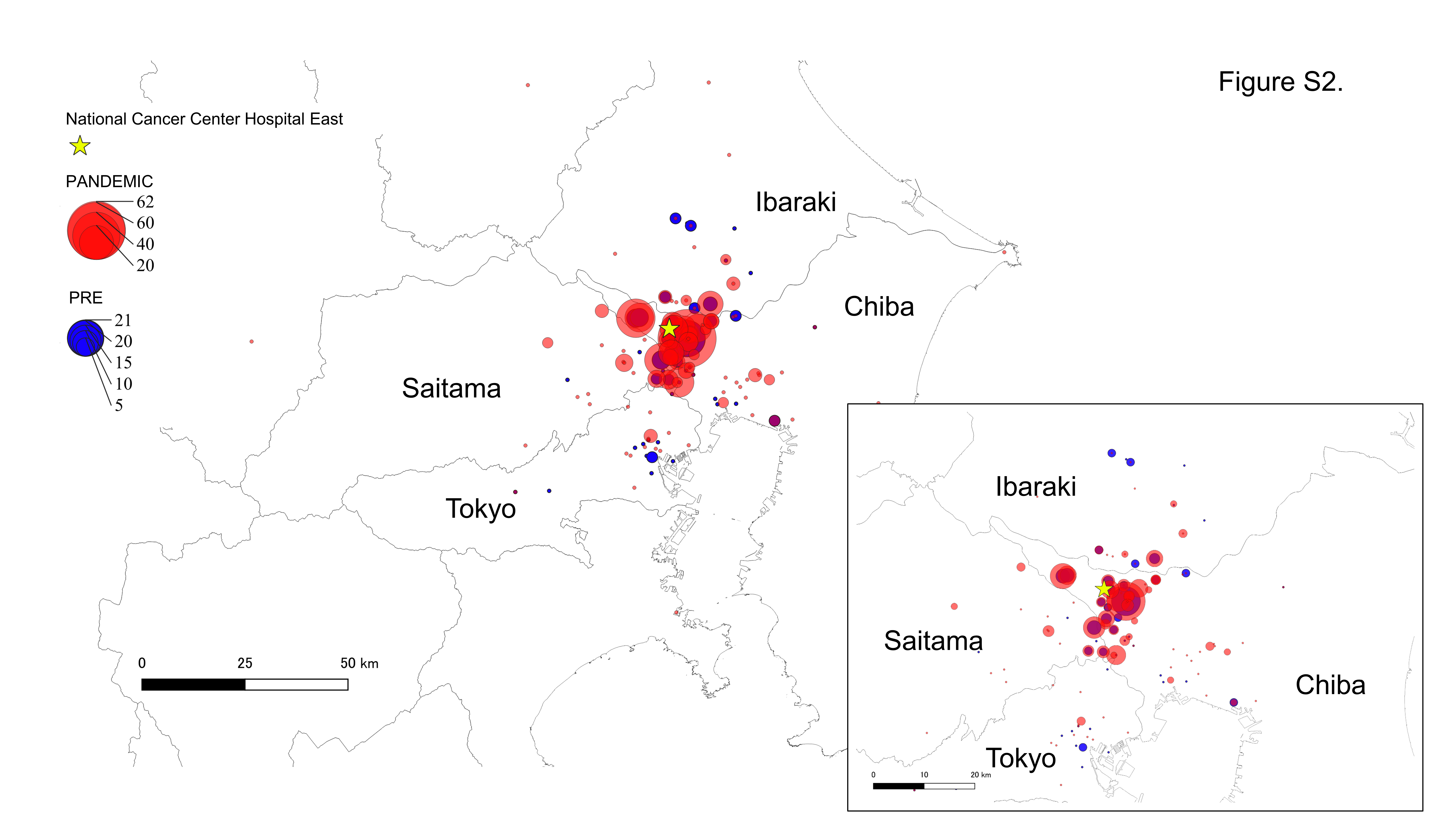

Supplement: Supplementary file 3 — Figure S2. [file CAM4-12-12095-s002.tif]

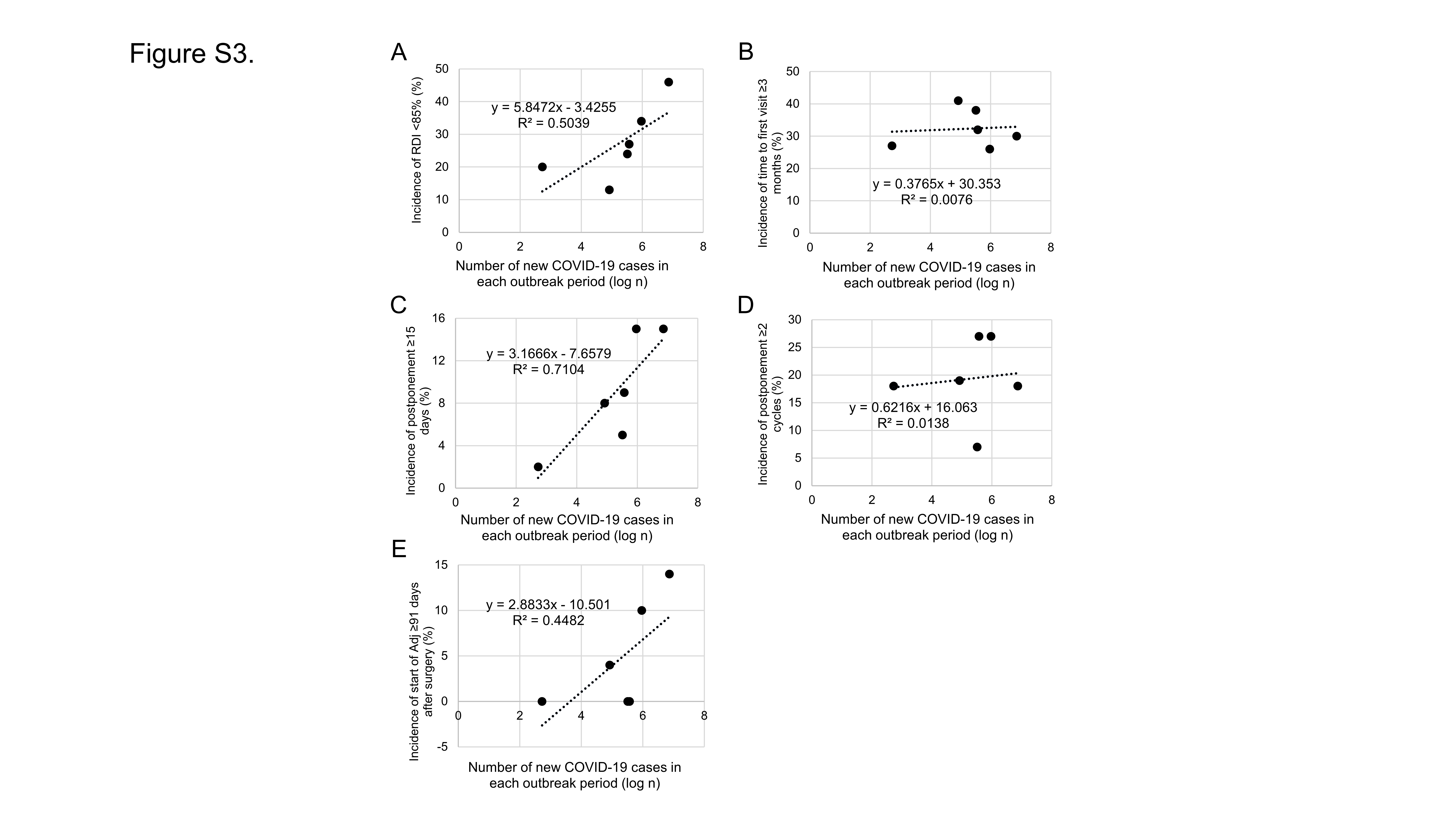

Supplement: Supplementary file 4 — Figure S3. [file CAM4-12-12095-s001.tif]
